# Evaluation of an Organisational Intervention to Promote Integrated Working between Health Services and Care Homes in the Delivery of End-of-Life Care for People with Dementia: Understanding the Change Process Using a Social Identity Approach

**DOI:** 10.5334/ijic.2426

**Published:** 2016-06-03

**Authors:** Sarah Amador, Claire Goodman, Elspeth Mathie, Caroline Nicholson

**Affiliations:** Research Associate, Marie Curie Palliative Care Research Department, University College London, 6th Floor, Wing B, Maple House, 149 Tottenham Court Road, W1T 7NF, UK; Professor of Health Care Research, Centre for Research in Primary and Community Care, University of Hertfordshire, College Lane, Hatfield AL10 9AB, UK; Research Fellow, Centre for Research in Primary and Community Care, University of Hertfordshire, College Lane, Hatfield AL10 9AB, UK; NIHR Postdoctoral Research Fellow, National Nursing Research Unit, King’s College London, 4th Floor Room 2.49b James Clerk Maxwell Building, Waterloo, Waterloo, London SE1 8WA, UK

**Keywords:** appreciative inquiry, complex interventions, dementia, end-of-life, long-term care settings, process evaluation, social identity

## Abstract

In the United Kingdom, approximately a third of people with dementia live in long-term care facilities for adults, the majority of whom are in the last years of life. Working arrangements between health services and care homes in England are largely ad hoc and often inequitable, yet quality end-of-life care for people with dementia in these settings requires a partnership approach to care that builds on existing practice.

This paper reports on the qualitative component of a mixed method study aimed at evaluating an organisational intervention shaped by Appreciative Inquiry to promote integrated working between visiting health care practitioners (i.e. General Practitioners and District Nurses) and care home staff. The evaluation uses a social identity approach to elucidate the mechanisms of action that underlie the intervention, and understand how organisational change can be achieved.

We uncovered evidence of both (i) identity mobilisation and (ii) context change, defined in theory as mechanisms to overcome divisions in healthcare. Specifically, the intervention supported integrated working across health and social care settings by (i) the development of a common group identity built on shared views and goals, but also recognition of knowledge and expertise specific to each service group which served common goals in the delivery of end-of-life care, and (ii) development of context specific practice innovations and the introduction of existing end-of-life care tools and frameworks, which could consequently be implemented as part of a meaningful bottom-up rather than top-down process.

Interventions structured around a Social Identity Approach can be used to gauge the congruence of values and goals between service groups without which efforts to achieve greater integration between different health services may prove ineffectual. The strength of the approach is its ability to accommodate the diversity of service groups involved in a given area of care, by valuing their respective contributions and building on existing ways of working within which practice changes can be meaningfully integrated.

## Introduction

In the United Kingdom approximately a third of people with dementia live in long-term care facilities for adults [1]. In England, it is estimated that 50% of these providers are care homes that provide personal and social care without on-site nursing [2]. These homes rely on primary health care and linked community physicians (i.e. General Practitioners – GPs) for access to medical care, community nursing (i.e. District Nurses – DNs) and specialist services [3]. Working arrangements between health services and care homes in England are largely ad hoc and often inequitable [4,5]. There is increasing recognition that effective health care interventions in these settings require a partnership approach to care that builds on existing practice [6].

The critical need for integrated working between health practitioners and care home staff is brought to the fore when considering care for residents with dementia approaching the end of life. Median length of stay of care home residents before they die has been found to be just under two years [7], however, difficulties of prognostication in dementia mean that when someone is actively dying is difficult to recognise [8,9,10]. Evaluative studies of interventions to improve end-of-life care in care homes have identified certain prerequisites for the successful implementation of end-of-life tools and frameworks in these settings. These include the cooperation of GPs with care homes [11] and the confidence of GPs in care home staff, for example, to make informed decisions as to when anticipatory medications could and should be administered [12]. Difficulties arise between care home staff, GPs, multi-disciplinary team members and families when there are competing accounts of who should lead care decisions at the end-of-life, misunderstandings, communication difficulties and different attitudes towards death and dying [12]. For example, utilisation of end-of-life frameworks that require staging residents’ illness, may prove difficult for care home staff who care for residents over a period of months or years [13] and who may as a result be reluctant to “think in these terms” [11] or experience difficulties in regarding dying as a “normal process” [14]. Quality end-of-life care in care homes is likely to be predicated on shared recognition that a resident is dying and concordance of end-of-life care values and goals among health care practitioners, care home staff and families [14]. Less well understood are the mechanisms by which greater cooperation between professionals and care staff from different organisations and disciplines can be achieved to improve and sustain end-of-life care.

This paper reports on the qualitative component of a mixed method study aimed at evaluating an organisational intervention to promote integrated working between visiting health care professionals (i.e. GPs and DNs) and care home staff. The intervention was developed as part of the “Evidence-Based Interventions in Dementia – End-of-Life” (EVIDEM-EoL) study, itself part of a larger five-year research programme to develop evidence-based interventions for older people with dementia from diagnosis through to end of life [15].

### Development of the project

EVIDEM-EOL was a study in two phases. In Phase One, we tracked the care received by 133 older people with dementia across six care homes in the East of England over eighteen months. A comprehensive description of Phase One methods and results are published elsewhere [15,16]. Overall, results highlighted existing capability and expertise in end-of-life and dementia care among visiting health care practitioners and care home staff respectively, but strong evidence of parallel working between both. All six homes received feedback and summaries of the Phase One findings and were invited to participate in the intervention phase. In Phase Two we implemented an organisational intervention broadly based on an Appreciative Inquiry approach, to promote integrated working between health services and care homes. Results from the quantitative assessment of the effectiveness of the intervention in terms of service utilisation and associated costs yielded promising results, specifically, a significant drop in post intervention hospital costs attributable to a decrease in ambulance call-outs and unplanned hospital inpatient stays among residents with dementia who participated in both phases of the study. Phase Two methods and results of the quantitative assessment and economic evaluation of the intervention are published in detail elsewhere [15,17]. This paper focuses on findings from the qualitative evaluation of the intervention, which uses a Social Identity Approach [18] to elucidate the mechanisms of action [19] that underlie the intervention, and to understand how these worked to effect changes in the delivery of end-of-life care for older people with dementia reported following the intervention.

## Methods

Methods presented here provide a summary of (i) the intervention approach and design, which were shaped by Appreciative Inquiry, (ii) methods for data collection and (iii) the theoretical framework (i.e. the Social Identity Approach) that guided the evaluation.

### Intervention approach

Appreciative Inquiry (AI) is an organisational development tool [20] that has been used in a variety of health care settings with participants from a diverse range of agencies, organisations and/or stakeholder groups (see Trajkovski et al. [21] for a recent review). Outcomes tend to include action and guidance plans [22,23], models [24], and suggestions and strategies for improvement [25,26] from which to inform future development of practice. Other less tangible outcomes resulting from the AI process include changes in terms of increased trust between stakeholders and organisations [22,27], more genuine and open communication [23,26,28] and overall “unleashing enthusiasm and cooperative capacity” [29]. AI was chosen as an appropriate framework for the intervention in as much as it has been likened to a “virtual or physical area designed for the creation of collective knowledge and the development of relationships” [30].

### Intervention design

AI is often conceptualised as a dynamic process in which participants are led through a cycle made up of four “phases”: Discovery, Dream, Design and Destiny [31]. The explicit objective of AI is formulated in terms of discovering what drives and sustains people and organisations when they are most effective. This is achieved through participants’ stories “of people working at their best” that are shared with the group. The Dream phase uses the stories from the Discovery phase to elicit common themes, ideas and understandings, with the aim of generating a shared image of the future. Once guided by a shared image of what might be achieved, members of the organisation find innovative ways (i.e. Design phase) to move the organisation closer to the ideal (i.e. its Destiny) [31]. Drawing on this framework, three AI meetings were planned over a period of six months, in each of the three homes that agreed to participate in Phase Two (see Table 1).

**Table 1:** Appreciative Inquiry meeting dates and approximate length.

Care home	CH1	CH5	CH6

Meeting one	25/01/2011 (01h10m)	03/02/2011 (01h30m)	19/01/2011 (01h10m)
Meeting two	15/03/2011 (02h00m)	24/03/2011 (01h45m)	14/03/2011 (01h45m)
Meeting three	28/06/2011 (02h10m)	29/06/2011 (01h45m)	20/06/2011 (01h10m)

All meetings were facilitated by a palliative care nurse researcher with experience in AI. Two researchers from the EVIDEM-EOL research team organised the meetings, provided information about the AI prior to the intervention, and summarised key themes and points for participants after each meeting. Table 2 summarises the main components of the intervention delivered across homes.

**Table 2:** Components of the intervention.

Components	Description	Prompts	Phase(s)

Appreciative conversations, AKA “Good Gossip”	Participants are invited to recount “*a successful story or positive memorable moment of working with others to provide end-of-life care for a resident dying with dementia*”. Common attributes, values, skills and abilities identified around providing good end-of-life care are highlighted and discussed	“What made the situation special? What was your contribution? How did you feel? How did others, either in the home or in the community help you?”	Discover, Dream
Development of future directed statements, AKA Common “Vision” for the home	Participants asked to imagine the care home 5 years on and their ideal for end-of-life care for people with dementia. Future-directed statements are also referred to as the participants’ common “Vision” for the care home. Participants encouraged to develop future-directed statements” into specific ideas for EOL innovations	What is different? What is going on in the home? Who is here? How have residents benefited?	Dream, Design
Resident Death Reviews (RDR)	An example of actual end-of-life practice within the home used to reflect on working practices and tease out the specifics of a potential innovation for end-of-life care. Review of events focused on the process of care, the alignment of strengths and the adjustment of practice (where necessary) that would help move towards the participants’ “Vision” for the care home.	All participants Prompted to discuss the resident death from the point of view of (i) the primary care doctor and/or District Nurse (ii) the care home staff and finally from (iii) the resident and family point of view.	Design

The intervention was designed to lead participants through the “Discovery” and “Dream” phases of AI to uncover existing capability in current end-of-life care practice through “Appreciative Conversations” and the development of a “Vision” for the home, i.e. a shared intent about the future of end-of-life care within the care home (see Table 2). In the “Design” phase, a review of actual practice preceding the death of a resident from within each home (i.e. resident death reviews) was used to reflect on working practices and to agree on potential changes in end-of-life care practice that would help move participants towards their “vision” for the care home.

### Intervention participants

AI meetings brought together care home staff and visiting health care practitioners (i.e. general practitioners and district nurses) involved in delivering end-of-life care for people with dementia. All members of staff across the three homes were invited to participate in the AI meetings. General practitioners and district nurses invited to participate in the intervention were the same as those recommended by care home managers to the research team (and who participated) in Phase One. Two GPs that participated in Phase One redirected the research team to another GP at the practice. Only one district nurse (i.e. attached to CH6) participated in both phases of the study. Two different DNs (i.e. attached to CH1 and CH5) were recommended to the research by care home managers for participation in Phase Two.

### Post-intervention exit interviews and follow up

Participants at the final round of AI meetings in each home were invited to take part in semi-structured post intervention exit interviews, to reflect on their experience of participating in the intervention and any perceived changes attributable to the intervention. Members of the research team were also, through ongoing work in the area of care home research, in a position to make field notes collecting evidence of sustained changes in working practices through exchanges with care home staff in the months following the intervention.

### Evaluation

Several approaches to explaining what promotes cooperative behaviour between groups have been proposed from within the social psychological study of intergroup relations [32,33]. A recent review of the literature examining health care groups has identified the “Social Identity Approach” or SIA [18] as a useful theoretical framework for understanding and overcoming “silo working” and divisions in healthcare. SIA defines social identity as derived from a sense of belonging to a positively valued group, and proposes that how we see ourselves and others in terms of social categories (i.e. “us” and “them”) affects our perceptions, attitudes and behaviour. Group identities are defined by specific norms, values, worldviews (i.e. identity “content”) that are meaningful and important to group members; and these are used to guide intergroup behaviour that can be either positive (e.g. cooperation) or negative (e.g. discrimination) depending on the context [33]. We can be led to cooperate with another group when encouraged to view their attitudes and/or behaviours as consistent with our own, that is, through a process of identity mobilisation. Haslam et al.’s ASPIRe (“Actualising Social and Personal Identity Resources) model [34] further elaborates on this strategy by viewing organisational change efforts towards increased cooperative behaviour between groups, as best grounded in the development of a shared or “superordinate” identity that involves “crafting a sense of us” [35], which also recognises the specific contributions of valued subgroups. In this model, identity mobilisation and context change (where context refers to the external working conditions such as working practices that support a particular system of group relations) are the key drivers of system change. Kreindler and colleagues [18] best summarise the need for both mechanisms to be present for change to occur in highlighting that “without mobilisation of valued identities, attempts to impose context change may provoke identity threat and invite implementation failure; without changes to the real conditions under which people work, identity mobilisation may amount to ‘just another staff development workshop’” (p. 365) [18].

Facilitated meetings and exit interviews were digitally recorded, anonymised, transcribed and analysed thematically using NVivo [36] to organise and manage the analysis. Data was coded by SA for instances of social identity language [37] and specifically for statements relating to group boundaries, as well as characteristics associated to end-of-life care for people with dementia that group member considered important. SIA and the ASPIRe model in particular were then used as the organising framework to identify patterns and differences that were consistent with or contradicted the frameworks’ key assumptions. These were reviewed, refined and debated by all the members of the team. Intervention participants were not involved in this stage of the analysis, but were invited to participate in all dissemination events related to the study.

### Ethical approval

Phase Two of this study (REC reference: 10/H0502/55) received a favourable ethical opinion from the Southampton and South West Hampshire Research Ethics Committee (A) on 10 August 2010.

## Results

In this section, we provide a brief overview of the characteristics of the intervention homes and participants, to provide the reader with background information to aid in the interpretation of results. These are reported in full elsewhere [15,17,38]. Drawing on the SIA framework and the ASPIRe model in particular, we consider what the findings revealed about identity mobilisation and the development of a common identity between service groups involved in end-of-life care for people with dementia, and how the intervention worked to implement changes in practice through the development of intergroup relations in these care settings.

### Care home and participant characteristics

Three of six care homes that participated in Phase One of the Study agreed to participate in Phase Two. Of the three that did not participate in the intervention phase: (i) one did not feel that they could improve on their end-of-life care (ii) one did not respond to the invitation despite repeated follow-up by the research team and (iii) one retracted after initially agreeing to participate when the manager changed jobs. Table 3 summarises participating care home characteristics.

**Table 3:** Care home characteristics.*

Care Home	CH1	CH5	CH6

Provider type	Private not for profit	Private	Private
Number of beds	46	67	57
Number of dementia places	46	67	57
Location	Suburban	Rural	Rural
Building	Local authority	Conversion	Purpose built

*Source: CQC listings’ AQAA data and manager interviews.

All three GPs approached to participate in the intervention accepted, and attended all three meetings. All three DNs approached to participate also agreed but attended only the first round of AI meetings. CH1 and CH5 DNs were no longer attached to these homes after the first meeting due to wider service reorganisation, and subsequently dropped out. Care homes involved staff as they were able. Care home managers and their deputies participated in all meetings except in CH5 where the manager attended only two before changing jobs. Exit interviews were conducted with visiting health care practitioners (n = 4) and care home staff (n = 5) that participated in all three meetings, up to four months post intervention. Characteristics of care home staff and the visiting health care practitioners that took part in the intervention are summarised in Table 4. Among care home staff, length of time working at the care home ranged between 15 months and 20 years. All members of staff had received training in dementia care. All members of staff had received some form of palliative care and/or end-of-life training either in-house or as part of vocational training. Overall, GPs had received little to no training in dementia or palliative care. All DNs had received training in palliative care. The frequency with which health care practitioners visited the homes was variable, ranging from weekly visits, to visits on request only.

**Table 4:** Characteristics of AI meeting participants.

Role (M/F)	Attendance	Professional qualifications Time since qualified (HCPs only)	Time at post in CH/visiting CH Frequency of visits (HCPs only)	Specialist Training Dementia Care	Specialist Training EOL Care

Care Home 1					

**Care Home Manager (F)**	All meetings	NVQ2, Btec NVQ4/Registered Manager’s award	7 years	Basic & intermediate Dementia Care Dementia Care Mapping Leadership in Dementia Care	In-house training with District Nurses
**Deputy Manager (F)**	All meetings	NVQ2 NVQ3 Contuning Care Supervisory Management Course	16 years	Certificate in Dementia Care Intermediate Dementia	In-house training with District Nurses
**General Practitioner (F)**	All Meetings	Lekarz, DCH, MRCPG 19 years	6 years/Once a week and on request	No	No
**District Nurse (M)**	1st meeting only	Registered Nurse 6 years	6 years/1 to 2 visits per month	No	Trust-run palliative care course
**Care Home 5**					

**Care Home Manager (F)**	1st and 2nd meeting only	NVQ4, RMA, AI, VI, PTLLS	Over 4 years	ASET Level 2 – Various Dementia certificates	Death, Dying & Bereavement
**Deputy Manager (M)**	All Meetings	NVQ Level 3 Leadership in Management course	15 months	Dementia level 2 award-currently working toward level 3	EOL e-learning Training
**General Practitioner (M)**	All Meetings	MBBS MRCP DRCOG MRCGP 10 years	6 months/Once a fortnight and on request	RCGP-run Dementia Management Course	No
**District Nurse (F)**	1st meeting only	Registered Nurse 11 months	11 months/1 or 2 visits per month	No	Trust-run training on syringe drivers & palliative care
**Care Home 6**					

**Care Home Manager (F)**	All Meetings	Registered Managers award PETALS (Preparing to teach in the lifelong sectors) NVQ Levels 2, 3, 4	6 years	Currently completing Dip in Dementia Care Qualified Dementia instructor	EOL training within NVQ
**Deputy Manager (F)**	All Meetings	NVQ Levels 2&3 PETALS (Preparing to teach in the lifelong sectors) Currently completing NVQ Level 4	3 years	Level 2 Dementia training	EOL training within NVQ
**General Practitioner (M)**	All meetings	MBBS, MROGP, DFSRM, DRCOG 3 years	Just under a year/On request only	No	Palliative Care Course
**District Nurse (F)**	All Meetings	Registered Nurse 33 years	Over 1 year/More than once a week	Dementia Patient Course with end of life module	Palliative Care Degree

### Identity mobilisation

Cooperative behaviour between groups can be driven by the recognition of a shared social identity rooted in common values and goals in a given area of care. The “Discovery and Dream” phases of the intervention uncovered certain characteristics of end-of-life care for people with dementia that were important to both groups (i.e. care home staff and visiting health care practitioners), including, for example, the value of involving family members throughout the different phases of end-of-life care up until the resident’s final moments. As both a GP and deputy care home manager (DCHM) observe:

“[When] there is mutual understanding on what is going to happen, so the family know, so it’s not a surprise, and that they are aware that the end could be coming and that it could be the end soon. I find that very positive when everybody knows” (GP-CH1-AIM1).

“[I]t was sort of, the most positive death that I’ve been involved with, in [all my] years of being here (…) everything that happened… I couldn’t have planned it better. The doctor was here, and the relative” (DCHM-CH6-AIM1).

The discovery phase also uncovered shared goals in the delivery of end-of-life care for people with dementia, and specifically, that residents be allowed to die in the care home should they so wish. In the following quote the deputy care home manager characterised this as providing family with the time and “space” to accompany their relative in their final days:

“People need to be made aware of that: that they are going to live in the care home and can stay until the end of their life. We can accommodate them if all parties are involved […] the family has more space and we can accommodate them to spend that last moment with their relative. Whereas if they are sent to hospital, there’s a time they have to leave, the hospital won’t accommodate that. Here we can create the space” (DCHM-CH5-AIM1).

Change in terms of greater cooperative behaviour is viewed as grounded in a shared social identity—built around shared values and common goals—but which also recognises the specific contributions of valued subgroups. Indeed, shared values and common goals highlighted above did not preclude perceived differences between subgroups as to when and how groups engaged with family and the type of input each group felt was within their range of expertise to provide. However, the AI process allowed these differences to be framed as valued contributions by both groups in the attainment of common goals. In CH1, the GP made explicit the value of the care home’s knowledge of the resident and their family, and how this informed and was integral to end-of-life conversations:

“I think [the care home staff] are fundamental to the conversation [about end-of-life wishes] (…) they could change the way things go, when it actually comes to the crunch (…) they know the family, they know the situation, they can say “the family wanted this”” (GP-CH1-AIM1).

The importance of care home staff’s role in all aspects of end-of-life care was similarly expressed by the GP in CH6, who highlighted care home staff’s often greater experience in dementia and palliative care:

“Prior to that, I haven’t really been involved in any deaths with dementia […] I actually found the care home to be a reassuring presence, in the sense that they know the drill, they are used to dealing with a lot of elderly residents. They’ve seen a lot [more] people die with dementia than I have and so in that sense, it was good to have someone else [there] with that experience, to sounds things off, you know? So that was good” (GP-CH6-AIM1).

CH6 was the only home in which the DN was retained throughout the AI intervention and unsurprisingly, the one in which the potentially valuable contribution of the district nursing service to end-of-life care was fully explored. In this home, the care home manager raised the question of what DN involvement would like beyond specific tasks such as wound care:

“We’d only really [call the DN] say if we’d got a pressure sore, something nursing needed […] You don’t even know… you don’t actually know what you’re asking for [in terms of palliative care if] you’re not asking of nursing input as such” (CHM-CH6-AIM2).

Further exchange led to shared recognition of the value in increasing district nursing involvement in end-of-life care, and implementation of “District Nurse Coffee Mornings” in the care home (for care staff and DNs to meet socially and talk about approaches to care) as a way to integrate the district nursing team into the wider staff group, and compensate for service changes in the district nursing team that were perceived to undermine working relationships.

### Context change

In addition to the development of a common group identity that recognises and values different groups’ respective expertise, SIA and the ASPIRe model in particular views context change, that is, in the real conditions under which people work, as a key in terms of greater cooperative working between groups. Allowing the resident to die in the care home, was found to be associated with practical challenges in managing sudden deterioration in these residents’ health and symptoms such as breathlessness, which could lead to callouts to emergency and Out-of-Hour (OOH) services. These challenges were highlighted and further explored through the resident death reviews (RDR – see again Table 2).

### Implementing tools to discuss end-of-life wishes of residents with family members

In CH1, the RDR focused on the particular case of a resident, who had died unexpectedly in an ambulance en route to hospital. For the manager and deputy there had been no clear indication that the resident was dying; both also highlighted the care home’s repeated experience of residents with dementia for whom recurrent illnesses (e.g. infection) were regularly treated with successful outcomes. Further discussion in this home led to recognising the importance of preparing, albeit tentatively, for end of life, to enable residents to die in their preferred place of care. As a result, staff chose to focus on developing their skills in this area using existing resources to discuss end-of-life wishes with family and carers developed by the Dying Matters coalition [39], which were shared with participants by the research team. In addition, the GP also changed the pattern of her visits, to allow time to review overall practice and general issues with the care home manager in addition to specific patient cases.

### Implementing tools to decide treatment plans

The RDR in CH5 examined the case of a resident for whom cardio-pulmonary resuscitation had been performed en route to hospital, where the resident eventually died, leading participants in this home to engage in more specific discussions around Do Not Attempt Resuscitation (DNACPR) orders. In this home, the GP made explicit how input from care home staff could trigger thinking about advanced care planning and appropriate documentation, and how care home staff and GPs could work together to prepare for end of life. Further discussion led the GP to suggest undertaking a review of all residents in close collaboration with care staff, to determine which, if any, were at a stage where discussions with the family may be appropriate regarding medical treatment plans including DNACPR.

### Implementing tools to manage services with no prior knowledge of the resident

In addition to the implementation of DN and care staff coffee mornings mentioned above, the resident death review led participants in CH6 to plan for how they could involve OOH GP services in end-of-life care, and draw on care staff expertise in providing a comprehensive history of the resident including any end-of-life wishes, to help OOH GPs to correctly assess the situation and deliver the most appropriate care. Together they developed an “OOHs Information Sheet” to be completed before calling OOH services. This highlighted key points of information about a resident that would help an OOH doctor judge the urgency of the situation and whether an emergency ambulance callout could be avoided or delayed until a doctor visited or not.

### Identity mobilisation and context change

SIA views change in terms of greater collaborative working as most likely to occur when both mechanisms (i.e. identity mobilisation and context change) are present. The following observations from the GP in CH1 illustrate how recognition of similar goals and values—building blocks of a common group identity—was perceived to facilitate routine interaction and give freedom to discuss end-of-life issues with care home staff:

“It’s important to share that we actually have the same goals, that we think the same things are important, it enables easier conversations on a daily basis, because we know where we’re all coming from” (GP-CH1-AIM3).

“Now that has happened you know, we talk, we have different relationship thanks to that […] even those quick interactions when we see each other, we could talk about … feel much more free to talk about the issues” (GP-CH1-EXIT).

In CH1 as in other homes, group identity was rooted in a shared view of the care home as a place where residents would likely die, and by extension, a place within which dying could be acknowledged and discussed. This in turn allowed prompts for discussing end-of-life wishes to be meaningfully introduced into every day practice:

“I think that meetings really helped us to focus on a bit more on [end of life] (…) it helps to focus on what we are doing [here]. Because people are coming here, are here for … we know that they, eventually, they will pass away anyhow (…) that is probably what we should be planning for (…) I know I used some of the pointers that were sent in with regard to talking about end of life (…) It was the resident herself and she did have dementia, however she was quite happy to talk about this, the subject of end of life (…) I was surprised actually” (DCHM-CH1-EXIT).

For the GP, a shift in how the care home setting was perceived was likewise accompanied by adjustments in advanced care planning practice including anticipatory prescribing:

“My focus has changed … always thinking whether the “just in case box” is required and making sure that the families’ views are known to everybody, and I know what the family wishes are” (GP-CH1-EXIT).

The intervention also fostered recognition of the respective contributions by care staff and GPs, and how these groups could work together to ensure residents be supported to die in their preferred place of care. Implementation of DNACPR policy in CH5 built on established ways of working that placed a high value on the contribution of care home staff to end-of-life decision-making. It also served to stimulate the construction of a group identity superseding those of “a carer and a doctor” within which completion of unified DNACPR forms that require joint review could be meaningfully integrated:

“The communication with [the DCHM] is no longer doctor-carer, ‘you do this, I’ll do that’ (…) the family [get] the impression that ‘this is just one body talking to me, rather than a carer and a doctor’ – basically just resonating that we think the same. (…) [I say] ‘I think at the moment we should just look at making sure she’s comfortable’ and you’ve got the carer there nodding [along] with you (…) saying ‘I’ll give you some information.’ (…) That definitely I don’t think would have happened without these meetings” (GP-CH5-AIM2).

The following two quotes from manager and deputy at CH5 respectively, illustrate how this also led to increased respect of each other’s roles, as well as increased trust leading to greater confidence among care home staff in ensuring compliance with end-of-life care plans:

“We’re confident to speak to the GP, to have that discussion. We’re not sort ‘oh goodness, we’ve got to phone the GP again’ (…) We know that there is somebody there listening and not dismissing what we’re saying. We’ve really got a good relationship and a lot of respect for each other’s roles now” (CHM-CH5-AIM2).

“I [can say] ‘no, the decision is [for the resident] to stay here [at the care home], be made comfortable’. From my point of view, when I look at it, I am confident, I know that the doctor [is involved] and he will back me up” (DCHM-CH5-AIM3).

In CH6 recognition of the contribution of care staff in providing a comprehensive history of the resident, allowed for the development of a tool to engage with OOH services in CH6, but also and perhaps more importantly in this home, to more immediate changes in terms of cooperative working between GPs, DNs and care home staff, for example in the exchange of personal phone numbers tied to greater inclination to call on others’ expertise:

“I just feel the communication is better with the care homes and especially with the GPs. We seem to speak to each other more don’t we? They now have our personal mobile number rather than having to go through one number. We talk to each other more” (DN-CH6-AIM3).

“I think I probably find it easier to talk about what is going on with either care staff or the district nurses. I probably seek help earlier. I probably seek others’ opinions more” (GP-CH6-EXIT).

Ultimately, the battery of practice changes triggered by the intervention supported recognition of valued contributions of subgroups, and active reconstruction of a common group identity from which to effect further changes in practice.

## Discussion

In summary, the AI intervention supported integrated working by fostering recognition of a shared social identity that had previously not existed, between care home staff and health care practitioners, rooted in shared values and common goals in the delivery of end-of-life care for people with dementia. The intervention also promoted recognition of existing knowledge, attributes, and expertise of subgroups, which could support common goals in the delivery of care. These included care staff’s expertise in dementia care, in-depth knowledge of the resident and unique relationship with family members, general practitioners’ expertise regarding medical treatment options, and district nurses’ expertise in palliative care. Finally, the intervention supported the development of context specific innovations in working practice (i.e. regular review meetings between care home staff and general practitioners, district nurse coffee mornings at the care home, OOHs information sheet), and the introduction and tailoring of existing end-of-life care tools and frameworks (i.e. prompts for discussing end-of-life wishes, DNACPR policies), which could reinforce a common group identity harnessing existing and specific expertise, from which to sustain collaborative working in the support of people with dementia at end-of-life.

AI is an organisational development tool that uses a co-design approach to create new ideas and images [20] and a “collectively desired future” [31] that reflects the range of views within a group [22]. Although variety of principles [40] have been put forward as underlying AI practice, none can usefully be called upon to help articulate intergroup processes and how they supported activities known to be key to integrated working observed throughout the AI. The SIA and ASPIRe model specifically achieves this by delineating mechanisms of action that underlie system transformation and making clear propositions of how these mechanisms work together to effect change allow us to reframe the “Dream” and “Design” phases of AI in terms of identity mobilisation and context change respectively. The framework also promotes greater understanding of how both components of the intervention reinforce each other to promote greater cooperation between services and actual change in the delivery of care.

AI is not unique in achieving change in end-of-life practice in care homes. Evaluative studies of end-of-life frameworks in care homes have highlighted a change in attitudes about dying among care home staff and overall change in end-of-life culture, which can improve end-of-life care [11,14,41]. The complementary mechanisms of identity mobilisation and context change can promote greater understanding how this is achieved, when considering practice changes in end-of-life care as an impetus for the construction of a common group identity based on shared views and common goals in this area of practice. Other studies investigating end-of-life care in care homes, have found working practices characterised by “active communication” [42] and “ongoing discussion” [43] between visiting health care professionals and care home staff, to be a fundamental feature of the delivery of high-quality end-of-life care in these settings. They have also identified the need to sustain the initial impact of cross organisational interdisciplinary working [44].

Practice changes developed as part of the AI process were not novel, but by grounding these in the development of a common group identity, the process of implementation was transformed into a meaningful bottom-up rather than top-down process. End-of-life tools and frameworks are best conceived here as relational as much as they are practice tools, which harness existing capability among service groups in the service of common goals. We found that deceptively small changes in terms of co-operative behaviour (exchange of personal numbers, seeking other people’s opinion, tokens of appreciation) were arguably critical in mitigating the unpredictability inherent to end-of-life care trajectories of older people with dementia and sustaining the use of tools and communication to support end-of-life care in these homes.

This was a study with a small number of participants and care homes. Evidence supporting greater cooperative behaviour is largely based here on reported rather than observed changes although there is evidence to suggest that end-of-life documentation improved, and use of secondary care services was reduced [15,17]. Throughout the study, end-of-life care programmes, policies and frameworks (end-of-life training, increased hospice involvement, ongoing training for GPs and in-house training for staff) were being piloted and introduced in the health care service. This may have influenced interest and decision-making within the groups. However, the findings from Phase One suggested a poor uptake of these. The emphasis of the intervention on participation and collaboration arguably led to greater engagement with existing resources for advanced care planning. Finally, future research will need to examine the impact of the facilitator’s own views on end-of-life care and group membership(s), and how these may impact on identity mobilisation throughout the intervention.

## Conclusion

Interventions structured around a Social Identity Approach can be used to gauge the congruence of values and goals between service groups without which efforts to achieve greater integration between different health services may prove ineffectual. Context sensitive interventions such as these can also give a potentially meaningful place to service users–including for people living and dying with dementia, and their families—by incorporating their views, values and goals as well as existing relationships with service providers, into wider organisational development. The strength of the approach is its ability to accommodate the diversity of service groups involved in a given area of care, by valuing their respective contributions and building on existing ways of working within which practice changes can be meaningfully integrated.
